# Maternal smoking and the risk of still birth: systematic review and meta-analysis

**DOI:** 10.1186/s12889-015-1552-5

**Published:** 2015-03-13

**Authors:** Takawira C Marufu, Anand Ahankari, Tim Coleman, Sarah Lewis

**Affiliations:** Division of Epidemiology and Public Health, Faculty of Medicine and Health Sciences, University of Nottingham, Clinical Sciences Building 2, Nottingham City Hospital, Hucknall Road, Nottingham, NG5 1 PB UK; Halo Medical Foundation, Osmanabad, India; Division of Primary Care, Faculty of Medicine and Health Sciences, University of Nottingham, Nottingham, UK

## Abstract

**Background:**

Smoking in pregnancy is known to be associated with a range of adverse pregnancy outcomes, yet there is a high prevalence of smoking among pregnant women in many countries, and it remains a major public health concern. We have conducted a systematic review and meta-analysis to provide contemporary estimates of the association between maternal smoking in pregnancy and the risk of stillbirth.

**Methods:**

We searched four databases namely MEDLINE, EMBASE, Psych Info and Web of Science for all relevant original studies published until 31^st^ December 2012. We included observational studies that measured the association between maternal smoking during pregnancy and the risk of stillbirth.

**Results:**

1766 studies were screened for title analysis, of which 34 papers (21 cohorts, 8 case controls and 5 cross sectional studies) met the inclusion criteria. In meta-analysis smoking during pregnancy was significantly associated with a 47% increase in the odds of stillbirth (OR 1.47, 95% CI 1.37, 1.57, p < 0.0001). In subgroup analysis, smoking 1-9 cig/day and ≥10 cig/day was associated with an 9% and 52% increase in the odds of stillbirth respectively. Subsequently, studies defining stillbirth at ≥ 20 weeks demonstrated a 43% increase in odds for smoking mothers compared to mothers who do not smoke, (OR 1.43, 95% CI 1.32, 1.54, p < 0.0001), whereas studies with stillbirth defined at ≥ 24 weeks and ≥ 28 weeks showed 58% and 33% increase in the odds of stillbirth respectively.

**Conclusion:**

Our review confirms a dose-response effect of maternal smoking in pregnancy on risk of stillbirth. To minimise the risk of stillbirth, reducing current smoking prevalence in pregnancy should continue to be a key public health high priority.

**Electronic supplementary material:**

The online version of this article (doi:10.1186/s12889-015-1552-5) contains supplementary material, which is available to authorized users.

## Background

Smoking in pregnancy is a major public health problem in the developed countries [1]. The World Health Organisation (WHO) predicts that this will reach ‘epidemic’ proportions in developing countries in the near future [1]. Within the current challenging economic climate in many countries, smoking in pregnancy imposes a significant burden on population health and resources, and is associated with a range of poor outcomes for both mother and child, such as ectopic pregnancy, miscarriage, placental abruption, preterm birth and low birth weight [2,3]. The harmful effects of tobacco smoke exposure in pregnancy can be avoided [4] and it is one of the most prevalent modifiable risk factors for adverse pregnancy outcomes [5,6].

In many developed countries, the rates of smoking in pregnancy have been declining over recent decades [7] with current prevalence estimates between 10% – 19% [8], and data from the UK suggesting that one out of eight women smoke throughout the pregnancy [9]. Moreover, this decline has not been consistent across all social classes; lower rates of decline have been noted across less advantaged socioeconomic groups [10]. Smoking in pregnancy in developed countries tends to be higher among women who have low income and poor education [11].

Stillbirth rates widely vary across low, middle and high-income countries [12-14]. The lowest rates have been observed in Finland with 2.0 per 1000 live births and in the developing world rates are much higher at more than 40 per 1000 live births in countries like Nigeria and Pakistan [6]. Two previous systematic reviews and meta-analyses have estimated the size of effect of smoking during pregnancy and the risk of stillbirth. A report by the United States Surgeon General [15] showed a relative risk of stillbirth of 1.2-1.8 within smokers versus non-smokers. The analysis was based on three studies [16-18] conducted in two countries, the United States and Sweden. Recent stillbirth statistics by Flenady et al [8] was based on a systematic review from high-income countries; and four studies were included in the meta-analysis of the association between any smoking in pregnancy and the risk of stillbirth yielding an odds ratio of 1.36 (95% CI 1.27, 1.46). Results from these published reviews were limited to literature from developed countries; yet stillbirth rates are much higher in developing countries, and it is therefore imperative to conduct a comprehensive review, which reflects the impact of smoking in pregnancy on stillbirth. We have conducted a systematic review and meta-analysis to provide contemporary estimates of the association between maternal smoking in pregnancy and the risk of stillbirth.

## Methods

A detailed electronic search was performed through four databases namely MEDLINE, EMBASE, Psych Info and Web of Science. All relevant published studies with sufficient data on maternal cigarette or tobacco smoking during pregnancy with the outcome of stillbirth were included. Due to the nature of the research objective, there were no randomised control trials (RCTs) identified, and observational studies (such as cohort design, case control studies and cross sectional surveys) were considered using the standard guidelines of Meta-analysis of Observational Studies in Epidemiology (MOOSE) [19]. All relevant studies published in the English language, up to 31^st^ December 2012 were included in the review. Case reports, non-English publications and those, which only involved passive or environmental smoke information, were excluded. We carefully considered studies where the study populations were similar to avoid duplication of cases; where studies were based on exactly the same population during the same time period, but there were differences in sample sizes, we selected the results from the publication with the larger sample size for inclusion in the meta-analysis, though those excluded from quantitative analysis were included in a narrative synthesis.

### Search strategy

We developed search terms based on medical subject headings (MeSH), free text words and words in the title or abstract. We combined search terms of exposure (smoking during pregnancy) with the outcome of the study (stillbirth). The MeSH terms used included combinations of the terms: stillbirth, maternal smoking, and pregnancy, also applied with special characters ($, *), wherever required. The study protocol was agreed within the team, however it was not published prior to commencement of the review. We also checked the reference list of all identified papers and the most recent similar reviews for any additional studies. A complete online search strategy along with search terms is attached (Additional file 1).

### Study selection and outcome definition

The electronic search was performed with a three-stage approach; first titles were screened by TM and AA, then abstracts were screened by two authors independently (TM and SL). For studies, which appeared to be eligible, full texts were obtained and reviewed independently by two authors who further performed data extraction independently on pre-piloted forms, and discrepancies were resolved by consensus. We also obtained full texts for those studies where a decision could not be made based on title and abstract. Data extraction forms included followings; study design, exposure validation, definition of exposure (smoking) and outcome (stillbirth), confounders adjustments, sample size and study location.

Since stillbirth is defined differently in different countries and different studies, stillbirth for this review was defined as fetal loss or death at 20 weeks gestation and above [20]; this included both early (20-28 weeks gestation) and late (after 28 weeks) stillbirth enabling inclusion of a wider range of international studies. Sensitivity analysis was done using alternative definitions of stillbirth:

- Stillbirth at ≥ 20 weeks of gestation
- Stillbirth at ≥ 24 weeks of gestation
- Stillbirth at ≥ 28 weeks of gestation

Where both unadjusted and adjusted measures were reported, the latter were extracted. Quality assessment was based on a Newcastle Ottawa Scale (NOS). Cohort and case control studies were awarded up to a maximum quality score of nine and cross sectional studies were given up to a maximum score of seven [21]. A definition of ‘high quality’, was given to cohort and case control studies with a score of 7 or above or cross-sectional studies with a score of 5 or above; the rest were deemed ‘low quality’ [21]. Each quality assessment (NOS) was conducted by at least by two authors independently and then findings were verified.

#### Statistical analysis

Meta-analysis was performed using Rev-Man 5.3 [22] with a random effects model. The I^2^ statistic was applied to calculate heterogeneity (I^2^ more than 75% was considered high heterogeneity, more than 50% moderate and 25% was considered as low heterogeneity) [22]. The odds ratio for the overall effect of smoking in pregnancy from each study was used, which was the main outcome reported in included literature. The ratio was calculated from the available data, wherever feasible. Studies included in the meta-analysis were further considered for subgroup analysis based on study quality, definition of stillbirth and cigarette consumption (1-9 cig/day and ≥10 cig/day). The funnel plot method was used to assess publication bias. Studies without statistical data presentation, and those where cases were potentially overlapping between publications, were considered in a narrative synthesis.

## Results

**Figure 1:**
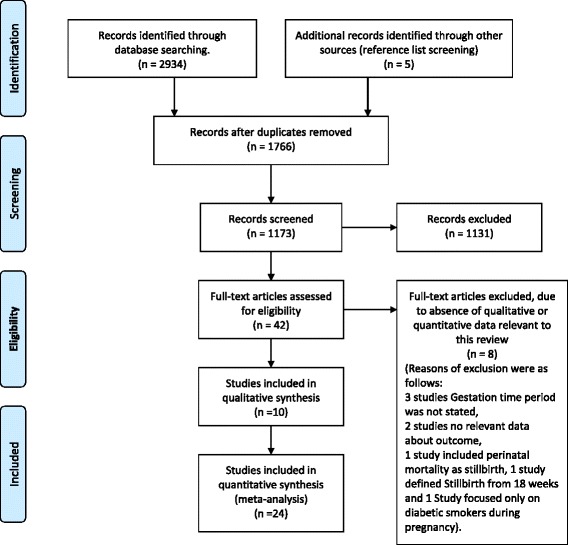
**Flow diagram of included and excluded studies.**

The initial database search produced 2,934 papers. We found 1,168 duplicate studies; meaning the same study was obtained from more than one of the four databases used in the review. After removal of duplicates, 1,766 study titles were screened, 94 were found eligible for abstract screening and 35 papers were considered for full text analysis. Out of 35 full texts sought, 34 were obtained within the available research timelines, and of these, 29 were included in the systematic review [12-14,16-18,23-45]. Eight full text studies, which did not have sufficient qualitative or quantitative data relevant to this review, were excluded [46-53]. An additional five relevant studies [54-58] were identified from references listed in the full text eligible studies. Details of the electronic search are explained in Figure 1. Out of the 34 included studies, 24 were included in the meta-analysis [12-14,17,23,24,26-29,31-41,54-56] and the other remaining ten in the qualitative synthesis [16,18,25,30,42-45,57,58]. One of the studies, [23] used two separate study designs, case control and bidirectional crossover methodology; therefore the study was considered in the meta-analysis separately as two observations resulting in 25 studies included in the quantitative synthesis. Two studies references [30,31] were derived from the same dataset (Missouri, USA) conducted in the same timeframe (1978-1997). Study [31] with a higher sample size was included in the meta-analysis and the other [30] was considered in the narrative synthesis. Four studies [16,17,25,29] used the same study population (Swedish National registry data) with differences in sample size and study methodology. Studies [17,25] had the same time frame, with study [17] having a higher sample size. Studies [16,17,29] had overlapping but not the exact dataset time frames. Study [16] time period (1983 – 1985) was exceeded by study 17 (1983-1989) which also had a higher sample size. Study [29] used the data for 1984 and 1991 for comparative purposes. To avoid having any woman’s data being counted twice in the meta-analysis, only the estimates obtained for 1991 data [29] were used in the meta-analysis. Two studies [17,29] were included in the meta-analysis whilst the other two studies [16,25] were included in the narrative synthesis only.

Included studies are further detailed in Table 1.

**Table 1:** **Table of included studies**

**Reference**	**Author year**	**Study type**	**Population**	**Location**	**Exposure: smoking during pregnancy and validity**	**Definition of stillbirth (outcome) and gestational period**	**Estimates**	**Factors adjusted for in individual studies if available**
***Studies included in meta-analysis***								
12	Wood et al (2009)	Cohort	158,502	Alberta Perinatal Health Program, 1991-2004 Canada	Smokers: Yes	Fetal death after at least 24 completed weeks of gestation.	Adjusted OR 1.04 (0.74-1.47)	Maternal age, diabetes, hypertension, previous caesarean section.
					Not validated			
13	Smith et al (2007)	Cohort	84,769	Scotland morbidity records 1991-2001, UK	Smokers: Yes	Fetal death after at least 24 completed weeks of gestation.	OR for smoking was calculated 2.15 (95% CI 1.74-2.65)	Age, deprivation, height, BMI, Marital Status.
					Not validated			
14	Hogberg and Cnattingius (2007)	Cohort	526,691	Sweden – Swedish National study 1983-2001- Participants with two consecutive pregnancies.	Smokers: Yes	Fetal death after at least 28 completed weeks of gestation.	OR for smoking in both Pregnancies 1.35 (1.15-1.58). 1-9cig/day OR 1.16 (0.92-1.46) ≥10 cig/day 0R 1.55 (1.17-2.04). OR for smoking in the 1^st^ pregnancy only OR 1.02 (0.79-1.30), 1-9 cig/day OR 1.13 (0.9-1.39), ≥10 cig/day OR 1.14 (0.87-1.51). OR for smoking in 2^nd^ pregnancy only 0.84 (0.55-1.26), 1-9cig/day OR 1.10 (0.89-1.35), ≥ 10 cig/day OR 1.45 (1.16-1.81).	Maternal age, education, cohabiting with infant’s father, mother’s country of birth, year of second delivery, inter-pregnancy interval and stillbirth in first pregnancy.
					1-9 cig/day, ≥10 cig/day.			
					Not validated			
26	Salihu et al (2008)	Case control	1,444,378	USA – Missouri 1978-1997.	Smokers: Yes	In-utero fetal death at least 20 week into gestation.	**a) Bidirectional case cross-over** OR 1.20 (1.03-1.39) 1-9 cig/day OR 1.16 (0.88-1.53) 10-19 cig/day OR 1.10 (0.90-1.24) ≥ 20 cig/day OR 1.21 (1.01-1.50),	Maternal race and age.
					1-9 cig/day,			
					10-19 cig/day,		**b) Case Control** OR 1.34 (1.26-1.43) 1-9 cig/day OR 0.94 (0.76-1.70) 10-19 cig/day OR 1.31(1.22-1.41) ≥20 cig/day OR 1.43 (1.31-1.54).	
					≥20 cig/day.			
					Not validated			
34***	Ahlenius and Thomassen (1999)	Cohort	94,270 in 1984 and 124,201 in 1991.	Sweden - Using the Swedish medical birth register in 1984 and 1991	Smokers: Yes.	Death of a fetus before or during delivery with a gestational duration of at least 28 weeks.	Combined (1984 and 1991) OR 1.37 (1.17 to 1.61)	Maternal age and parity.
					1-9 cig/day, ≥10 cig/day.		1-9 Cig/day OR 1.22	
							(1.00-1.48)	
					Not validated		≥10 Cig/day OR 1.62 (1.31-2.00)	
							1991 OR	
							1.47 (1.19 to 1.82)	
							>10 Cig/day OR 1.97 (1.51-2.59)	
36***	Aliyu et al (2007)	Cohort	1,436, 725	USA Missouri – Using Missouri maternally linked cohort data 1978 – 1997	Smokers: Yes	In utero fetal death at < = 20 weeks gestation	OR(originally presented as HRs) 1.43 (1.36-1.51)	N/A*
					1-9 cig/day, 10-19 cig/day, ≥ 20 cig/day.			
					Not validated			
37	Gray et al (2009)	Cohort	532,016	UK- Scotland, National study 1994-2003.	Smokers: Yes.	24-44 weeks’ gestation	OR 1.75 (1.61-1.91)	N/A*
					Not validated			
38	McCowan et al (2007)	Cohort	69,173	New Zealand - National Women’s	Smokers: Yes	Birth of a baby with no signs of life delivered at ≥	OR 1.33 (0.99-1.79)	Infant sex, maternal ethnicity, parity, marital status, maternal age at delivery, history of previous miscarriage, history of previous abortion, previous low-birth weight infant, previous C-section.
				Hospital, Auckland, New Zealand from 1993 to 2000	Not validated	20 weeks or with a birth weight of > 400 g if gestation was not known.		
39	Miller et al (2010)	Cohort	359,747	Canada – Ontario state 2004-2006	Smokers: Yes	Death of fetus at 20 weeks of gestation	Overall OR 1.58 (1.38-1.81)	Age, parity, multiple gestation
					Not validated			
40	Moshin et al (2005)	Cohort	433,227	Australia – New South Wales 1998-2002	Smokers: Yes	Fetal death at ≥ 20 weeks gestation or of 400 g or more birth weight.	Overall adjusted OR 1.17 (1.05–1.31) Unadjusted OR 1.05 (0.94–1.17).	Maternal age, maternal country of birth, socioeconomic status, maternal diabetes mellitus, and first antenatal care visit.
					Not validated			
17***	Raymond et al (1994)	Cohort	638,242	Sweden National study 1983-1989	Smokers: Yes	Fetus death at 28 weeks of gestation or more.	OR 1.4 (1.2-1.4)	Age, parity, pregnancy complications, hypertensive diseases, diabetes, placental complications and intrauterine growth retardation (IUGR).
					Not validated			
42	Reddy et al (2006)	Cohort	174,809	USA – 12 clinic centres and 19 hospital sites. 2002-2008.	Smokers: Yes	Fetal death at 23 weeks gestation and beyond.	OR (originally presented as HRs) 1.57 (1.21-2.02)	N/A*
					Not validated			
43	Sutan et al (2010)	Cohort	541,811	UK- Scotland, National study (NHS Hospital)	Smokers: Yes	Late fetal death from 20 weeks gestation.	OR 1.64 (1.46-1.84)	Non stated
					Not validated			
44	Tuthill et al (1999)	Cohort	16,047	Wales – All Wales Perinatal Survey 1993-1995	Smokers: Yes	Mortality from 20 completed weeks of gestation	OR 1.72(1.38-2.13)	Social class, infection, placental abruption, sudden infant death syndrome.
					Not validated			
45	Wisborg et al (2001)	Cohort	25,102	Denmark – Aarhust University Hospital 1989-1996.	Smokers: Yes	Delivery of a dead fetus occurring at 28 weeks or more gestation.	OR 1.9 (1.3-2.9),	Sex of child, parity, maternal age, education, alcohol, caffeine intake.
					1-9 cig/day, ≥10 cig/day.		1-9 cig/day OR 1.5 (0.9-2.4) ≥10 cig/day 1.8 (1.2-2.8)	
					Not validated			
46	Winbo et al (2001)	Cohort	1,412,747	Sweden – National study 1983 - 1995	Smokers: Yes	Death of fetus after competing 28 weeks of gestation.	OR stillbirths ≥37 weeks compared with live births 1.08 (0.96-1.22)	Non stated
					Not validated			
27	Ferraz and Gray (1991)	Case control	11,483	Brazil – Natal city and state of Rio Grande do Norte 1984-1986	Smokers: Yes	Stillborn cases weighing 500 g or more	OR 1.4 (1.0-2.0)	Non stated
					Not validated			
29	Dodds et al (2006)	Case control	494	Canada – Nova Scotia and Eastern Ontario 1999-2001	Smokers: Yes	Not specified - Death before delivery of a fetus weighing 500 g or more	OR (originally presented in HRs) Smoking during 1^st^ trimester 2.13 (1.23-3.64)	N/A*
					Not validated			
30	Efkarpidis et al (2004)	Case control	599	UK – Nottingham City Hospital 1991-1997.	Smokers: Yes	Definition of stillbirth not stated- pregnancies considered from ≥ 24 weeks	OR 1.2 (0.64-1.6)	Non stated
					Not validated			
31	Froen et al (2002)	Case control	598	Norway – Oslo 1986-1995.	Smokers: Yes	Intrauterine death before onset of labour of a fetus > =22 weeks gestation or > = 500 g body mass	OR calculated Using Stats 1.81 (1.02-3.15) 1-9 cig/day OR 1.0 (0.43-2.12), ≥10 cig/day 0R 3.04 (1.13-8.19)	N/A*
					1-9 cig/day, ≥10 cig/day.			
					Not validated			
32	Goy et al (2008)	Case control	510	Canada – Nova Scotia and Eastern Ontario 1999-2001.	Smokers: Yes	Not specified - Death before delivery of a fetus weighing 500 g or more	OR 2.06 (1.22-3.47)	Province of origin, age, inactivity during pregnancy, stillbirth in previous pregnancy, used fertility treatment
					Not validated			
33	Little and Weiberg (1993)	Case control	4667	USA	Smokers: Yes	Fetal death at 28 weeks or more gestation	OR calculated using Stata 1.29 (1.09-1.53)	N/A*
					1-19 cig/day,		OR 1-19 cig/day 1.3 (1.09-1.57), 20-29 cig/day OR 1.39 (1.06-1.82), ≥30 cig/day OR 0.67 (0.41-1.12).	
					20-29 cig/day,			
					≥ 30 cig/day.			
					Not validated			
47	Mishra et al (2005)	Cross-sectional	16,802	India - Survey conducted 98-99	Smokers: Yes	Delivery of a dead baby at > 28 weeks of pregnancy	OR 1.23 (0.92-1.64)	Cooking smoke, anaemia, BMI, education, standard of living, house type.
					Not validated		Unadjusted OR 1.60 (1.23-2.08).	
48	Robson et al (2006)	Cross-sectional	21, 880	Australia – New South Wales 1998-2003.	Smokers: Yes	Not stated	OR 1.65 (1.15-2.38)	Age, parity, medical/obstetric complications
					1-10 cig/day >10 cig/day.	20 weeks	1- 10 cig/day OR 1.16 (0.80-1.69) >10 cig/day OR 1.68 (1.17-2.41).	
					Not validated			
***Studies included in narrative synthesis***								
16***	Cnattingius et al (1988)	Cross-sectional	262,582	Sweden National Study – Using Swedish medical birth registry covering more than 99% of all births in Sweden. 1983-1985	Smokers: Yes	Late fetal death – still birth occurring at 28 weeks of gestation or later	OR (originally presented as RR) 1.37 (1.20-1.57)	N/A*
					1-9 cig/day,		1-9 cig/day 1.32 (1.12-1.55)	
					≥10 cig/day.		≥10 cig/day 1.45 (1.21-1.75)	
					Not validated			
28***	Walles (1994)	Case control	202	Sweden – 5 centres (Boras, Helsingborg, Karlskrona, Kristianstastad and Lund) 1983-1989.	Smokers: Yes	Definition of stillbirth not stated, pregnancies considered	OR calculated using Stata. OR 1.18 (0.62-2.26)	N/A*
					1-10 cig/day,		1-10 cig/day OR 1.33 (0.58-3.09) >10 cig/day 1. 04 (0.43-2.50)	
					≥10 cig/day.	≥ 28 weeks		
					Not validated			
35***	Aliyu et al (2008)	Cohort	1,436,628	USA Missouri – Using Missouri maternally linked cohort data 1978 – 1997.	Smokers: Yes.	In utero fetal death at < = 20 weeks gestation.	OR (originally presented as HRs) 1.48 (1.40-1.56)	N/A**
					< 35 years old, ≥ 35yers old. Not validated		< 35 years smokers1.43 (1.35-1.51)	
							≥35 years Smokers OR 2.4 (2.06-2.80)	
49	Kleinman et al (1988)	Cohort	362,261	USA Missouri 1978-1983	Smokers: Yes	(Fetal mortality) Fetal death at 20 weeks or more.	OR Primiparas	N/A**
					< 1 pack/day ≥1 pack/day.		<1 pack/day 1.36(1.16-1.59) ≥1 pack/day 1.62 (1.34-1.97)	
					Not validated		Multiparas	
							<1 pack/day 1.21 (1.06-1.38) ≥1pack/day 1.15(0.99-1.34)	
50	Raatikainen et al (2006)	Cohort	25,591	Finland-Kuopio University Hospital 1989-2001.	Smokers: Yes.	Fetal death was defined as intrauterine death before	No estimates	N/A**
					Not validated			
						22 weeks of pregnancy or 500 g fetal weight.		
51	Aliyu et al (2010)	Cohort	633,849	US Missouri – Using Missouri maternally-linked cohort data files 1978 – 1997.	Smokers: Yes	In utero fetal death at > = 20 weeks gestation.	Results HRs,	N/A**
					Not validated		<15 years 3.3 (1.4-7.8), 15-19 years 1.7 (1.5-1.9), 20-24 years 1.5 (1.4-1.6)	
							No sufficient data to calculate Odds ratios.	
52	Bai et al (2000)	Cohort	7138	Australia- Liverpool hospital Sydney March 1996 to June 1998	Smokers: Yes	Not Clear (births occurring at less than 20 weeks gestation age exclusion criteria).	Results presented in Percentages. No odds ratios and no sufficient data to calculate OR.	N/A**
					Not validated			
53	Huang et al (2000)	Cohort	84,294	Canada – The Royal Victoria Hospital in Montreal 1961-1974 and 1978-1996.	Smokers: Yes	Fetal deaths occurring before labour without evidence of significant fetal, maternal or placental pathology weighing 500 g or more.	No estimates	N/A**
					Not validated			
18	Schramm (1997)	Cross-sectional	176,843	USA – Missouri 1978-1990	Smokers: Yes Not validated	Fetal death greater or equal to 20 weeks gestation.	Results in RR no sufficient data to calculate OR	N/A**
							Smoking in 1^st^ and not in 2^nd^ pregnancy RR 1.11, Not smoking in 1^st^ but in 2^nd^ pregnancy RR 1.6.smoking in both pregnancies RR 1.19.	
54	Salihu et al (2004)	Cross-sectional	7,792,990	USA – 1995-1997.	Smokers: Yes	Intrauterine fetal demise after 20 weeks of gestation.	Estimates in Hazard ratios not enough data to calculate OR.	N/A**
					Not validated		≥ 40 years 2.71 (1.88-3.91), 30-39 years 1.58 (0.96-2.60), 20-29 years 1.41 (1.16-1.71)	

OR – Odds Ratio, HR – Hazard Ratio, RR – Risk Ratio.

N/A* - Original results were presented in HR/RR, OR was calculated using original data.

N/A** - Studies included in descriptive synthesis only.

*** - Studies using same dataset.

Swedish National registry data set ref 16: 1933-1985, 17 and 28 : 1983-1989, ref 34: 1984 and1991.

US – Missouri data set ref 35 and 36.

Out of the 34 eligible studies (Table 1), eight were case control studies [23-28,54,55], five cross sectional [16,18,40,41,45] and twenty-one were cohort studies [12-14,17,29-39,42-44,56-58]. Fourteen were conducted in Europe, [13,14,16,17,25,27,29,32,36-39,43,54], four in Australia [34,41,56,58], fourteen in North America [12,18,23,26,28,30,31,33,35,42,44,45,55,57], one in Asia [40] and one in South America [24]. The largest sample size observed was 7,792,990 [45] with the smallest being 202 [25]. Ten studies [13,16,25,26,30-32,35,54,55] did not present their results with odds ratios but had sufficient data to calculate the ratio using STATA 12, which was further used in the meta-analysis. Seven studies [18,42-45,57,58] did not present estimates or sufficient data to calculate an odds ratio; therefore they were included in the narrative synthesis. Based on NOS scale, overall study quality was moderately satisfactory. Ten cohort studies [12,14,17,32,35-39,42] and four case control studies [23,24,54,55] were considered of high quality with seven or more points, and only two cross sectional studies [16,45] were of high quality having five or more points. Scores ranged from 2 to 9 and the median score was 6.

### The type of exposure

Eleven studies clearly defined the level of exposure (smoking) [14,16,17,25,29,31,33,34,38,54,56]. Most of the studies used categories of 1-9cig/day and ≥10 cig/day to categorise participants according to the number of cigarettes or packs consumed daily, but the Robson study [41] used slightly different categories. Seventeen studies did not define smoking according to level of cigarette consumption [12,13,24,26-28,30,32,35,36,40,41,43,45,55,57,58] but according to the smoking status (yes/no). Fifteen studies collected exposure information during pregnancy [12-14,16,17,27,29,30,32,33,38,42,43,54,56] and the rest of the studies [18,23-26,31,34,35,39-41,44,45,55,57,58] obtained the information after delivery. None of the studies reported biochemical validation of smoking status such as salivary cotinine assessments.

### Data collection on outcome

The definition of stillbirth varied across studies. Twenty-one out of thirty-four studies included stillbirth from early gestational age ≥ 20 weeks [18,23,30,31,33,34,36,37,41-43,45,56-58], ≥22 weeks [54], ≥23 weeks [35], and four studies used birth weight to estimate the gestational week of stillbirth (Birth weight ≥ 500 g, [24,26,28,44]. The rest of the studies used late gestational ages as the cut off values for stillbirth, ≥24 weeks in four studies [12,13,27,32], ≥28 weeks in nine studies [14,16,17,25,29,38-40,55]. In all studies, outcome data was either obtained from medical or clinical records, medical data sets or birth registry records.

Fourteen studies included in the quantitative synthesis adjusted for one or more confounders (Table 1). Maternal age was the most common adjusted factor. Five studies adjusted for socioeconomic status (SES) including education [13,14,37,38,40]. Other factors that were adjusted for include parity, BMI, ethnicity/race, infant sex, perinatal care and marital status, alcohol, caffeine intake and cohabitation. Four studies [24,27,36,39] did not adjust for any confounders and six studies [26,31,32,35,54,55] had OR calculated therefore considered as not adjusted for any confounders. Factors adjusted in cohort studies varied widely. Several studies [12,17,28,34,37,41,56] considered one or more medical conditions such as pregnancy complications, diabetes mellitus and hypertension as possible confounders.

### Meta-analysis of maternal smoking and risk of stillbirth

**Figure 2:**
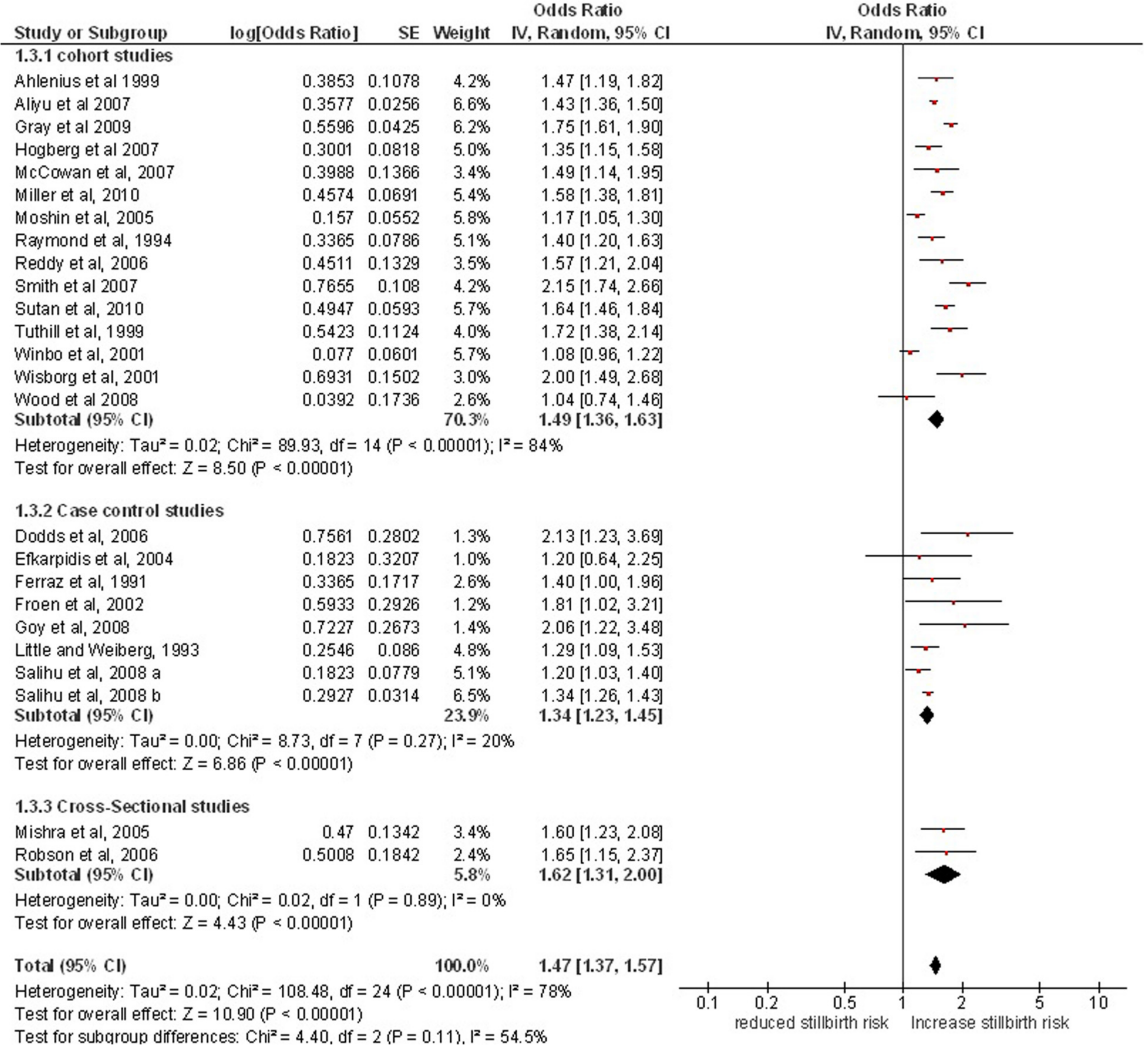
**Maternal smoking in pregnancy and the risk of stillbirth.**

In meta-analysis of all 25 studies (Figure 2), smoking during pregnancy was associated with a 47% increase in the odds of stillbirth (OR 1.47, 95% CI 1.37, 1.57, p < 0.0001) with an overall moderate heterogeneity (I^2^ = 79%). There was no significant difference in the size of this estimate between study designs (p = 0.11); the odds of stillbirth were increased by 34% in relation to smoking in pregnancy in case-control studies (OR 1.34, 95% CI 1.23, 1.45, p < 0.0001, 8 studies), 49% in cohort studies (OR 1.49, 95% CI 1.35, 1.64, p < 0.0001, 15 studies) and 62% in cross-sectional studies (OR 1.62, 95% CI 1.31, 2.00, p < 0.0001, 2 studies). Results were more heterogeneous for cohort studies than other study types.

#### Subgroup analysis by study quality

Results from high quality score studies showed that women who smoke during pregnancy, are 41% more likely to have a stillbirth compared to women who do not smoke during pregnancy (OR 1.41, 95% CI 1.28, 1.55, p <0.0001, 14 studies). Low quality score studies showed that women who smoke during pregnancy were at 49% increased odds of stillbirth compared to women who do not smoke during pregnancy (OR 1.49, 95% CI 1.33, 1.67, p <0.0001, 11 studies). There was no significant difference between the two subgroups (p = 0.44).

#### Subgroup analysis by categories of cigarette consumption

**Figure 3:**
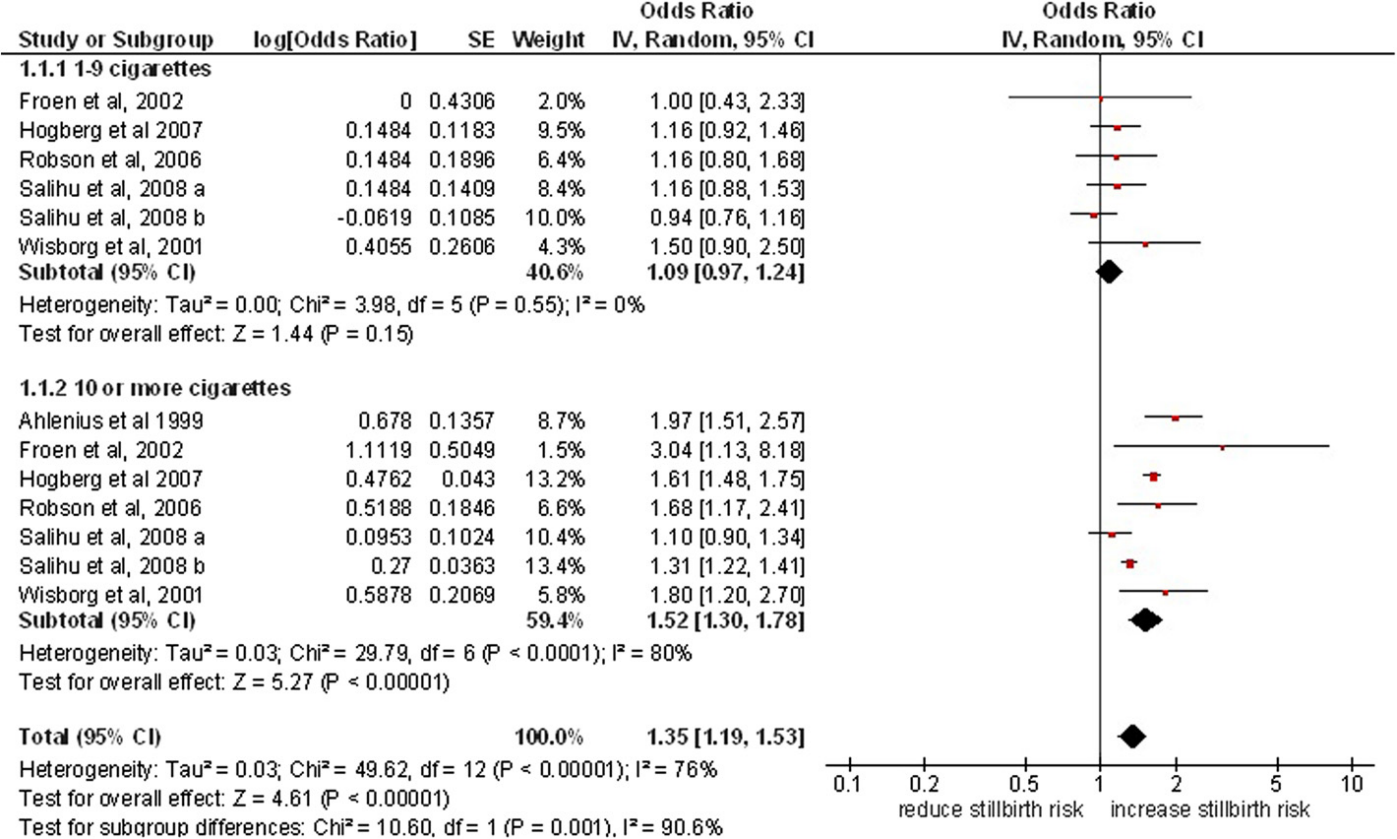
**Stillbirth risk according to the amount of daily cigarette consumption.**

Seven studies (14, 26 a-b,31,34,45,48) were included in this subgroup analysis, with consumption categorised as 1-9 cig/day and ≥10 cig/day (Figure 3). One study [29] presented OR values for the ≥10 cig/day category only. A study by Robson et al [41] used slightly different categories; 1-10 cig/day and >10 cig/day. This study was included in this analysis with odds ratios for the 1-10 cig/day, and > 10 cig/day categories used for the 1-9 cig/day and ≥ 10 cig/day groups respectively. Further two studies by Salihu et al (2008a) [23] and Salihu et al (2008b) [23] had a classification of 1-9 cig/day, 10-19 cig/day and ≥ 20 cig/day. There was no sufficient raw data to calculate the OR for ≥10 cig/day. Therefore these studies have been included in this analysis conservatively using the ORs from the 10-19 cig/day categories (which is a lower value than the ≥ 20 cig/day value) for the ≥ 10 cig/day subgroup. Most of the studies included in this subgroup analysis (4 out of 7) used this categorisation (1-9 cig/day and ≥10 cig/day), thus the same was applied in the subgroup analysis. Meta-analysis showed that smoking 1-9 cig/day was associated with an 9% increased odds of having a stillbirth compared to women who do not smoke in pregnancy (OR 1.09, 95% CI 1.09, 1.24, p = 0.55, 6 studies), whilst smoking ≥10 cig/day was associated with a 52% increase in odds of stillbirth (OR 1.52, 95% CI 1.30, 1.78, p < 0.0001, 7studies); the results for these two subgroups differed significantly (p = 0.001).

#### Subgroup analysis by gestation of stillbirth

**Figure 4:**
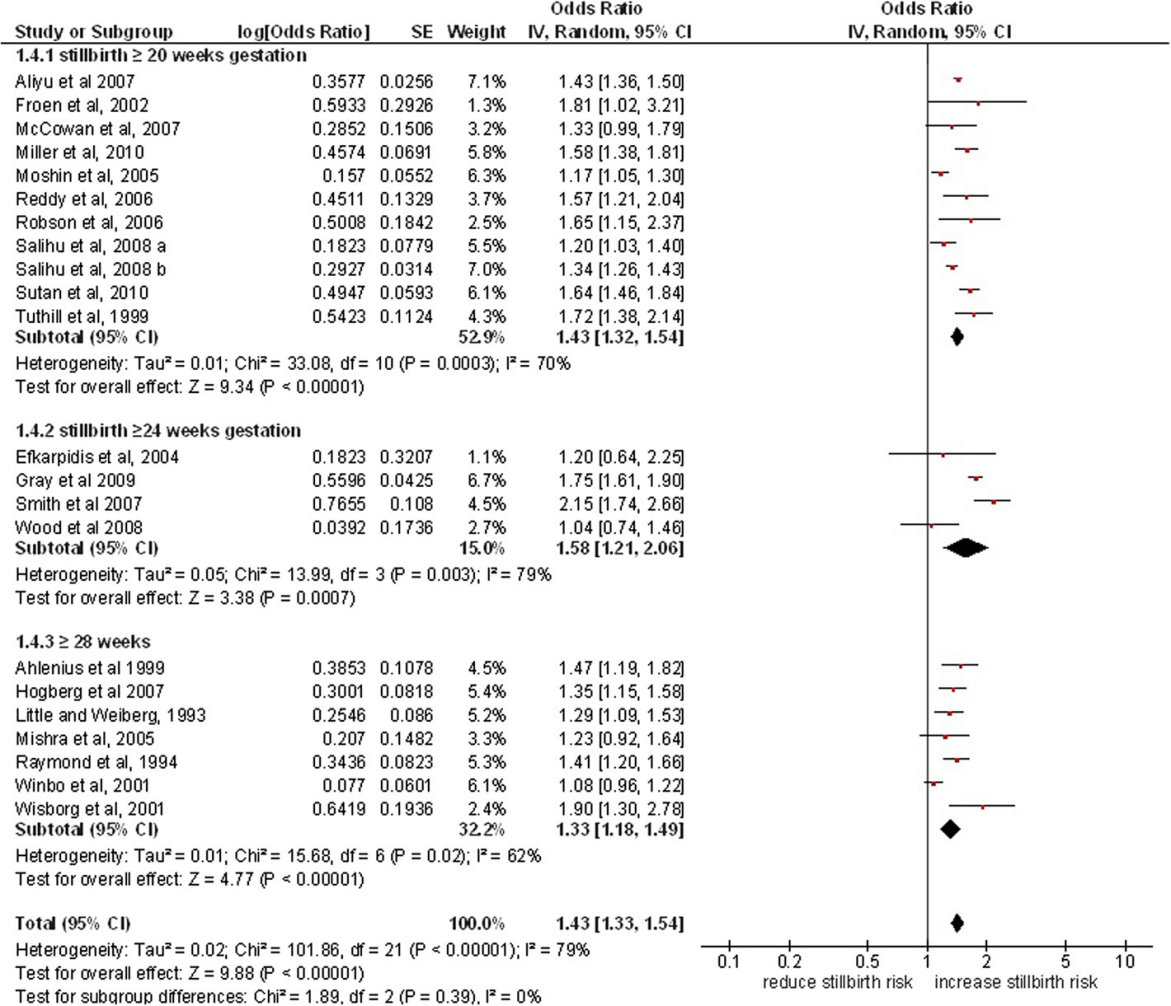
**Maternal smoking and the risk of stillbirth according to the gestation period.**

Twenty-two studies were included in this subgroup analysis (Figure 4). The meta-analysis of studies defining stillbirth at ≥ 20 weeks demonstrated a 43% increase in odds for smoking mothers compared to mothers who do not smoke, (OR 1.43, 95% CI 1.32, 1.54, p < 0.0001, 11 studies), whereas studies with stillbirth defined at ≥ 24 weeks showed 58% increase in odds (OR 1.58, 95% CI 1.21, 1.2.06, p < 0.0003, 4 studies) and in pooled estimates of studies with stillbirth defined at ≥ 28 weeks, the odds was increased to 33% (OR 1.33, 95% CI 1.18, 1.49, p < 0.02, 7 studies). There was no significant difference in the odds ratios for these different subgroups (p = 0.39).

#### Subgroup analysis by study location

Only two studies were conducted in developing countries [24,40]. Both studies support evidence from research conducted in developed countries concluding that women who smoke during pregnancy are at a risk of stillbirth. A study [40] demonstrated a very marginal difference showing those women who smoke during pregnancy are at least 40% at risk of experiencing a stillbirth (OR 1.40, 95% CI 1.0, 1.96), similar to the pooled odds ratio of studies conducted in North America (OR 1.39, 95% CI 1.29, 1.50) P value < 0.0001, 9 studies) and Europe (OR 1.55, 95% CI 1.36, 1.78, P value < 0.0001,11 studies).

#### Narrative synthesis

Seven studies [18,42-45,57,58] presented their results in other than odds ratio and there was no sufficient data to calculate the odds ratio, and three studies [16,25,30] were derived from same study population as [17,31] respectively. Six of the studies [16,18,30,42,45,57] observed an association between maternal smoking in pregnancy and the risk of stillbirth but the other four [25,43,44,58] observed no significant differences. Two studies [16,42] observed a dose related response with high risk ratio of stillbirth in mothers who smoke ≥10 cig/day compared to 1-9 cig/day and those who smoke ≥1 pack/day in contrast to <1 pack/day respectively. Three studies (51, 35 54) showed the risk of stillbirth to be higher in young mothers <15 years old (HR 3.3, 95% CI 1.4, 7.8) and also in older mothers (≥35 years, HR 3.2, 95% CI 2.2, 4.5) (≥40 years, HR 2.71, 95% CI 1.88, 3.91) respectively. A study from Sweden suggested that placental abruption is likely to be common in smokers [25].

### Publication bias

The publication bias was assessed visually using a funnel plot developed by Rev Man 5.3 [22]. The plot was symmetrical indicating a low risk of publication bias (Additional file 2).

## Discussion

This study provides a comprehensive review of the current evidence and estimates of the effect of maternal smoking on the risk of stillbirth. It suggests, women who smoke during pregnancy have a 47% increased risk of stillbirth and that the risk of stillbirth is more at higher levels of cigarette consumption (Table 2). This effect does not vary according to the gestation at which still birth occurs, however, comparisons between studies would be easier if future work adopted the definition of stillbirth recommended by the WHO [20].

**Table 2:** **Result summary: maternal smoking in pregnancy and the risk of stillbirth**

**Assessment criteria**	**Odds ratio**	**95% CI**
*i) Overall pooled Results from main meta-analysis (25 studies)*	1.47	1.37-1.57
*ii) Subgroup analysis by daily amount of cigarettes consumption*		
1-9 cig/day (6 studies)	1.09	0.97-1.24
≥ 10 cig/day (7 studies)	1.52	1.30-1.78
*iii) Subgroup analysis by gestation period at which stillbirth occurred*		
Stillbirth ≥ 20 weeks (11 studies)	1.43	1.32-1.54
Stillbirth ≥ 24 week (4 studies)	1.58	1.21-2.06
Stillbirth ≥ 28 weeks (7 studies)	1.33	1.18-1.49
*iv) Subgroup analysis by geographic location*		
North America (9 studies)	1.39	1.29-1.50
Europe (11 studies)	1.55	1.36-1.78
Asia (1 study)	1.23	0.92-1.64
South America (1 study)	1.40	1.00-1.96
Australia (3 studies)	1.29	1.07-1.55
*v) Subgroup analysis by quality*		
High score (14 studies)	1.41	1.28-1.55
Low score (11 studies)	1.49	1.33-1.67

### Strengths and limitations

We implemented a comprehensive search strategy with strict adherence to the protocol, and results were presented in accordance with MOOSE guidelines [19]. Search strategy, data extraction, analysis and quality assessment was performed independently by authors and findings were confirmed within the team. There was no evidence of publication bias. However, the review has some limitations. We could not obtain one study within available timelines [59], and due to limited data presentation, seven studies [18,42-45,57,58] were considered in the narrative synthesis. Secondly, only English language studies were considered mainly for practical purposes and the available timeframe.

Heterogeneity was explored using a variety of subgroup analyses; a high level of heterogeneity was found amongst cohort studies and the pooled estimate derived from these should be treated with some caution. This could be partially attributable to the different factors controlled for in the cohort studies as explained previously. The overall heterogeneity (I^2^ = 77%, Figure 2) was higher mainly due to cohort studies, however in case of case control studies (I^2^ = 20%) and cross-sectional studies (I^2^ = 0%), very low levels of heterogeneity was observed. However, this estimate was consistent with synthesised estimates derived from studies with other designs and this consistency suggests it is likely to be valid. It appears that the effect could differ by age [24,30,38,45,57], but data on maternal age was not available in included studies. Study quality was found to be satisfactory.

### Synthesis

The most recent systematic review by Flenady et al [8] on maternal smoking and the risk of stillbirth, focusing on high-income countries, reported a 36% increase in the odds of stillbirth (OR 1.36). The review considered cohort and case control studies published between 1998 and 2009 and excluded those which did not control for confounding factors, hospital based studies and took a slightly different approach to dealing with duplicate studies. Acknowledging the difference in the approach, our study is in agreement with the results from Flenady’s et al [8] review, and showed a 46% increased risk of stillbirth (OR 1.46 95% CI 1.36, 1.55). We included 25 studies in our main meta-analysis, conducted in both developing and developed countries making our findings generalisable. Our review has also analysed the available data according to the definition of stillbirth in gestational weeks, number of cigarettes and study quality, which were not reported in the previous published literature. The review also provides strong evidence that the risk is higher at higher levels of cigarette consumption, indicating a dose related response relationship, outlined in some of the included studies [15,38,42].

The consistency in the size of the effect observed in this study throughout different subgroup analyses and across different study designs, each with their own biases and strengths and limitations, as well as the consistency across studies of differing quality, suggests that this is a true estimate of the size of effect. Further support is obtained from studies where mothers change in smoking behaviour from one pregnancy to another. Hogberg and Cnattingius [14] suggested that mothers who quit smoking from first to second pregnancy reduced their risk of stillbirth to the same level as non-smokers in both pregnancies, while those who smoked in both pregnancies had a 35% increased risk of stillbirth (OR 1.35) compared to non-smokers.

Our results also provide evidence that the effect of maternal smoking in pregnancy on risk of stillbirth is not dependent on the definition of stillbirth or on the stage of pregnancy at which it occurs. Moreover we did not ascertain, at what point during pregnancy the effect of the exposure is occurring as most studies evaluated the exposure at one point in time only. Previous studies [26,38] suggested that the timing of exposure may influence the risk of stillbirth and in particular [38] suggested that those who quite early in pregnancy may have similar risk to non-smokers. Future studies of this association should measure exposure at different stages of gestation. There are important potential confounders for the effect of maternal smoking on risk of stillbirth, the most important ones are SES, maternal age, maternal weight and medical comorbidities, majority of studies have adjusted for these. Only two studies did not adjust for any confounders [36,43] with no further information.

The exact pathophysiology of fetal exposure to tobacco smoke is not entirely understood, however based on available evidence, possible mechanisms have been conceptualised [30]. Nicotine has been known to cause narrowing of the placental vessels [60,61], which coupled with reduced prostacycline synthesis [62], resulting in increased vascular resistance and consequently impairing blood supply to the fetus [63]. Carbon monoxide in tobacco smoke also reduces fetal oxygenation by forming carboxy-haemoglobin in turn interfering with oxygenation transfer [64]. The correlation between smoking and stillbirth is likely to be explained through increased prevalence of placental complications and fetal growth restriction [14]. The resultant physiological effect of these changes increases the risk of fetal morbidity (small for gestation age and preterm birth) and subsequently may lead to fetal death [30]. Therefore, there is strong biological plausibility for smoking in pregnancy causing stillbirth [46,65].

Recent Cochrane reviews have concluded that behavioural interventions can be effective in helping women to stop smoking in pregnancy [66] even though there remains no evidence for the effectiveness of pharmacotherapy such as NRT in pregnancy [67]. The result of our study suggests that such interventions are important to reduce the risk of stillbirth.

## Conclusion

Our findings support that smoking greatly increases the risk of stillbirth. Smoking in pregnancy is an established cause of a range of pregnancy complications and poor pregnancy outcomes. Every opportunity must be utilised to record smoking status during pregnancy, to give advice and support, including necessary facilities to help women stop smoking. Although we have conducted a comprehensive review, most of the available evidence on the risk from smoking is from developed countries. Smoking is rapidly increasing in the developing world where stillbirth is a major problem; therefore along with focusing on research initiatives, it is important to invest efforts on educating women on the risks of smoking to their unborn child and providing smoking cessation support for pregnant women in these parts of world.

## Additional files

Additional file 1:
**Supplementary 1.** Sample search strategy used for identification of studies. Additional file 2:
**Funnel plot.**

## Abbreviations

NOS: New castle Ottawa scale
SES: Socioeconomic status
