# Supplementary material for: Maternal smoking and the risk of still birth: systematic review and meta-analysis
Source: BMC Public Health. 2015 Mar 13;15:239. doi: 10.1186/s12889-015-1552-5 (PMC4372174; doi:10.1186/s12889-015-1552-5)
Supplement: Additional file 1: — Supplementary 1. Sample search strategy used for identification of studies. [file 12889_2015_1552_MOESM1_ESM.pdf]

**Supplementary 1: Sample Search strategy used for identification of studies**

A thorough databases (Medline, Embase, Psych Info and Web of science) search, using the below proposed individual and combines search terms was carried out in sets. Medical Subject Headings (MeSH) thesaurus and free text terms and outlined combinations were amended accordance to the requirements of the database under interrogation, with an understanding that some databases do not use MeSH. It should also be noted that some search interfaces have limited functionality (for example those which support single line searches only or allow small numbers of search terms). In such circumstances initial searches were performed using broad maternal smoking exposure terms or foetal outcome measures.

**Search Terms**

**A**

1) Maternal

2) Mother*

3) Woman

4) Women

5) 1 or 2 or 3 or 4 or 5

**B**

6) Pregnan*

**C**

7) Smoking*

8) Tobacco

9) Cigarette*

10) Cessation

11) 11 or 12 or 13 or 14 or 15 or 16

**D**

12) Stillbirth*. ti,ab.

13) Still-birth*. ti,ab.

14) (Fetal adj death*). ti,ab.

15) (Foetal adj death*). ti,ab.

16) (Fetal adj loss*). ti,ab.

17) (Foetal adj loss*). ti,ab.

18) (Fetal adj mortality). ti,ab.

19) (Foetal adj mortality). ti,ab.

20) (Infan* adj mortality). ti,ab.

21) Antepartum loss*. ti,ab.

22) Antepartum death*. ti,ab.

23) Intrapartum loss*. ti,ab.

24) Intrapartum death*. ti,ab.

25) Perinatal loss*. ti,ab.

26) Perinatal death*. ti,ab.

27) (Pregnancy adj outcome*). ti,ab.

28) 13 OR 14 OR 15 OR 16 OR 17 OR 18 OR 19 OR 20 OR 21 OR 22 OR 23 OR 24 OR 25 OR 26 OR 27 OR 28.

29) 5 AND 6 AND 11 AND 28

**<u>Search used of Web of Science</u>**

#5 #4 AND #3 AND #2 AND #1

DocType=All document types; Language=All languages;

#4 Topic=(stillbirth*) OR Topic=(still-birth*) OR Topic=(fetal death*) OR Topic=(foetal death*) OR Topic=(fetal loss*) OR Topic=(foetal loss*) OR Topic=(fetal mortality) OR Topic=(feotal mortality) OR Topic=(pregnanacy outcome*) OR Topic=(antepartum loss*) OR Topic=(antepartum death*) OR Topic=(intrapartum loss*) OR Topic=(intrapartum death*) OR Topic=(perinatal death*) OR Topic=(perinatal loss*)

DocType=All document types; Language=All languages;

#3 Topic=(smoking*) OR Topic=(tobacco) OR Topic=(cigarette*) OR Topic=(cessation)

DocType=All document types; Language=All languages;

#2 Topic=(pregnancy) OR Topic=(pregnant) OR Topic=(pregnan*)

DocType=All document types; Language=All languages;

#1 Topic=(mternal) OR Topic=(mother*) OR Topic=(woman) OR Topic=(women)

DocType=All document types; Language=All languages;
