# Supplementary figures and images for: Maternal smoking and the risk of still birth: systematic review and meta-analysis
Source: BMC Public Health. 2015 Mar 13;15:239. doi: 10.1186/s12889-015-1552-5 (PMC4372174; doi:10.1186/s12889-015-1552-5)

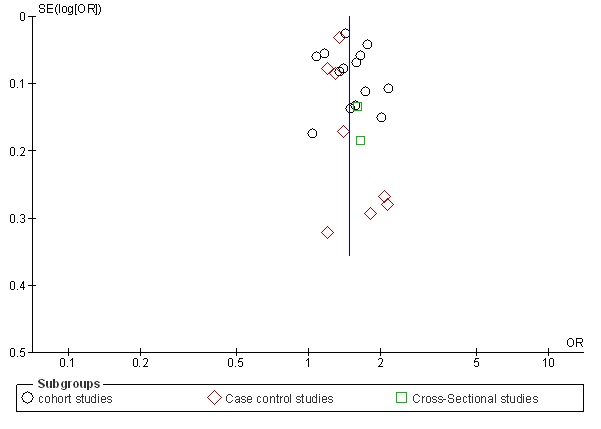

Supplement: Additional file 2: — Funnel plot. [file 12889_2015_1552_MOESM2_ESM.jpeg]
